# *Cunninghamella* spp. produce mammalian-equivalent metabolites from fluorinated pyrethroid pesticides

**DOI:** 10.1186/s13568-021-01262-0

**Published:** 2021-07-08

**Authors:** Mohd Faheem Khan, Cormac D. Murphy

**Affiliations:** UCD School of Biomolecular and Biomedical Science, University College Dublin, Belfield, Dublin 4, Ireland

**Keywords:** Biotransformation, cytochrome P450, ^19^F NMR, fungi, insecticide

## Abstract

*Cunninghamella* spp. are fungi that are routinely used to model the metabolism of drugs. In this paper we demonstrate that they can be employed to generate mammalian-equivalent metabolites of the pyrethroid pesticides transfluthrin and β-cyfluthrin, both of which are fluorinated. The pesticides were incubated with grown cultures of *Cunninghamella elegans*, *C. blakesleeana* and *C. echinulata* and the biotransformation monitored using fluorine-19 nuclear magnetic resonance spectroscopy. Transfluthrin was initially absorbed in the biomass, but after 72 h a new fluorometabolite appeared in the supernatant; although all three species yielded this compound, it was most prominent in *C. blakesleeana*. In contrast β-cyfluthrin mostly remained in the fungal biomasss and only minor biotransformation was observed. Gas chromatography-mass spectrometry (GC–MS) analysis of culture supernatant extracts revealed the identity of the fluorinated metabolite of transfluthrin to be tetrafluorobenzyl alcohol, which arose from the cytochrome P450-catalysed cleavage of the ester bond in the pesticide. The other product of this hydrolysis, dichlorovinyl-2,2-dimethylcyclopropane carboxylic acid, was also detected by GC–MS and was a product of β-cyfluthrin metabolism too. Upon incubation with rat liver microsomes the same products were detected, demonstrating that the fungi can be used as models of mammalian metabolism of fluorinated pesticides.

**Supplementary Information:**

The online version contains supplementary material available at 10.1186/s13568-021-01262-0.

## Key points

- *Cunninghamella* spp. biotransformed transfluthrin and β-cyfluthrin.
- New metabolites were detected by ^19^F NMR and GC–MS.
- Rat liver microsomes produced the same metabolites when incubated with the pesticides.

## Introduction

In contrast to the very small number of fluorinated natural products that are known, approximately 30% of agrochemicals contain fluorine (Ogawa et al. 2020). Fluorine’s attractiveness lies in its unusual properties of small atomic size, high electronegativity, high redox potential and strength of the carbon-fluorine bond. Thus, bioactive compounds that contain fluorine have enhanced characteristics in terms of metabolic stability, lipophilicity and enzyme/receptor binding. However, the increasing application of fluorinated compounds in agriculture, amongst other industries, has resulted in environmental pollution with these xenobiotics and their metabolic by-products (Murphy and Sandford 2015). The stability of the C-F bond, which is an advantage in certain circumstances, often means that complete biodegradation is not possible. Thus, organisms are exposed to these xenobiotics, or their dead-end metabolites, and may be subjected to their harmful effects, which are not fully understood (Murphy 2016).

Pyrethroids are widely used insecticides that are structurally related to naturally-occurring pyrethrins that are produced by *Chrysanthemum* spp. They are classified as type I or type II depending on whether they contain a cyano group in their structure (type II). Furthermore, several pyrethroids contain fluorine either as a fluoroaliphatic moiety (e.g., cyhalothrin) or fluoroaryl group (e.g., silafluofen), or both (tefluthrin). Although very effective, there is a high degree of concern surrounding the health and environmental effects of pyrethroid insecticides, in particular their effect on aquatic organisms (Hintzen et al. 2009; Yang et al. 2018).

It is important to have methods for understanding the metabolism of fluorinated xenobiotics in mammals, and whilst animal testing is one method that is widely used, there are practical and ethical issues that make it increasingly unattractive. Some microorganisms are effective models of mammalian xenobiotic metabolism, in particular species of the fungus *Cunninghamella*. These fungi have cytochromes P450 (CYP) enzymes that catalyse phase I (oxidative) reactions and conjugative enzymes capable of phase II reactions (Palmer-Brown et al. 2019a; Zhang et al. 1996). The most commonly used species for drug biotransformation studies are *C. elegans*, *C. echinulata* and *C. blakesleeana*. These fungi are known to catabolise fluorinated xenobiotics (Amadio et al. 2010) and non-fluorinated pesticides such as diazinon (Zhao et al. 2020) and fenitrothion (Zhu et al. 2017). In addition, the fungi can form biofilms that can be applied to the continuous production of drug metabolites (Bianchini et al. 2021; Souza et al. 2016) and dye decolorisation (Hussain et al. 2017).

**Fig. 1:**
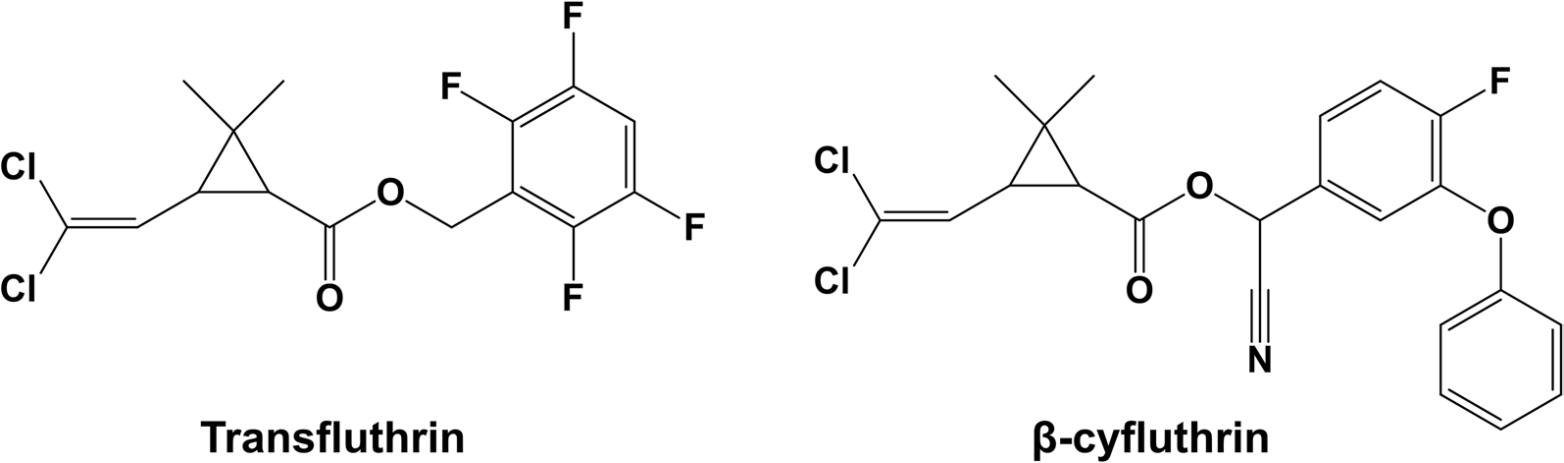
Structures of the fluorinated pyrethroid insecticides transfluthrin and β-cyfluthrin

Previously we investigated the biotransformation of the fluorinated pyrethroid cyhalothrin in planktonic and biofilm cultures of *C. elegans* (Palmer-Brown et al. 2019b). In this paper we describe the biotransformation of the related pesticides transfluthrin and β-cyfluthrin by *Cunninghamella* fungi and compare the metabolites produced to those found in experiments with rat liver microsomes. Both compounds belong to the pyrethroid family of pesticides, are found in the product Baygon and both are fluorinated (Fig. 1). A microbial method for producing mammalian-equivalent metabolites of these pesticides will enable convenient toxicity testing and improve decision-making for their disposal.

## Materials and methods

### Fungi, microsomes, insecticides and other reagents

*Cunninghamella blakesleeana* DSM1906, *C. echinulata* DSM1905, and *C. elegans* DSM1908 were acquired from DSMZ German Collection of Microorganisms and Cell Cultures GmbH and used for the present research. Rat liver microsomes were purchased from Sigma (Arklow, Ireland). The pyrethroid insecticides, transfluthrin (PESTANAL®) and β-cyfluthrin (PESTANAL®) were obtained from Sigma (Arklow, Ireland) and used for biotransformation studies. The culturing media, reagents and solvents used in the study were of analytical and molecular grade and purchased from Sigma (Arklow, Ireland) and Merck (USA), include Sabouraud dextrose broth (SDB), Sabouraud dextrose agar (SDA), dimethylformamide (DMF), methanol d_4_, ethyl acetate, *N*-methyl-*N*-(trimethylsilyl)trifluoroacetamide (MSTFA), and NADPH tetrasodium salt.

### Cultivation of *Cunninghamella *spp.

All the *Cunninghamella* species were cultivated according to the procedure described by Amadio et al. (2010). Briefly, the fungal mycelia were first grown on SDA plates (for 5 days at 28 °C) and the mycelium (with agar) was homogenised with 100 mL of autoclaved water to prepare inocula. To grow fungi in liquid cultures, inoculum (5 mL) was added to 45 mL autoclaved SDB media in 250-mL Erlenmeyer flask and incubated for 72 h at 28 °C and 150 rpm agitation. The grown fungi were then used for biotransformation experiments.

### Fungal biotransformation of insecticides

Transfluthrin, (TF) and β-cyfluthrin (BCF) were dissolved in DMF and introduced to the 72 h-grown cultures of *C. blakesleeana* (CB), *C. echinulata* (CE), and *C. elegans* (CEL) at a final concentration of 0.1 mg/mL. The cultures were incubated at 28 °C with 150 rpm agitation for 0, 24, 72 and 120 h. The fungal biomass was separated from aqueous fraction (or supernatants) by centrifugation at 9000 rpm for 10 min. Control experiments were conducted in which the fungi were incubated in the absence of pesticide.

Both culture supernatant and biomass were extracted using ethyl acetate. The supernatant was extracted twice with 50 mL ethyl acetate using 250-mL separating funnel, whereas the biomass was extracted with 100 mL ethyl acetate in 250-mL Erlenmeyer flask at continuous shaking at 150 rpm for 2 h. For sample preparation, the solvent was removed under reduced pressure using rotary evaporator and the residue was collected in 2-mL glass vials by re-dissolving in a small volume of ethyl acetate. For estimation of total biotransformation, extracts from supernatant and biomass were combined and analysed by ^19^ F NMR.

### ^19^F-NMR and GC–MS analysis

The samples were analysed by fluorine-19 nuclear magnetic resonance (^19^F NMR) spectroscopy and gas chromatography-mass spectrometry (GC–MS). For ^19^F NMR analysis, the samples were dried under N_2_ gas, re-dissolved in 700 µL methanol d_4_, and analysed using Varian 400 MHz spectrometer. For GC–MS analysis, the dried samples (under N_2_ gas) were silylated using 60 µL MSTFA at 100 °C for an hour; the final sample volume was adjusted to 0.5 mL by adding ethyl acetate. The silylated samples were analysed in the split mode (20:1) using 7890B N Agilent GC system. The instrument was equipped with a HP-5MS capillary column (30 m × 0.25 mm × 0.33 μm) and a 5977 A mass-selective detector. The oven temperature was initially set at 90 °C for 3 min then raised to 300 °C at 10 °C/min rate.

### CYP Inhibition experiment

To differentiate the CYP and non-CYP dependent biotransformation, 1-aminobenzotriazole was used as a CYP inhibitor. The 72 h-grown fungal cultures in the Erlenmeyer flasks were incubated with 3 mM 1-aminobenzotriazole and incubated at 28 °C and 150 rpm for one hour prior adding the insecticide. The supernatants and fungal biomass were extracted, silylated and analysed by GC–MS and ^19^F NMR as described above.

### Microsomal biotransformation of insecticides

As *Cunninghamella* spp. are recognised as fungal models of mammalian xenobiotic biodegradation owing to their ability to catabolise them in an analogous manner (Palmer-Brown et al. 2019a). Rat liver microsomes were used to investigate the metabolism of TF and BCF. Following the method of Obach et al. (1999), 15 mg/mL microsomes were incubated with 2 mM insecticides (dissolved in DMF), 2.5 mM NADPH and 3.3 mM MgCl_2_ in 0.7 mL sodium phosphate buffer (30 mM, pH 7.5) for 4 h at 37 °C and 120 rpm. The metabolites were extracted, silylated and analysed by GC–MS as described above. Control experiments in which microsomes were incubated in the absence of pesticide were also conducted.

## Results

### Monitoring biotransformation by ^19^F NMR spectroscopy

**Fig. 2:**
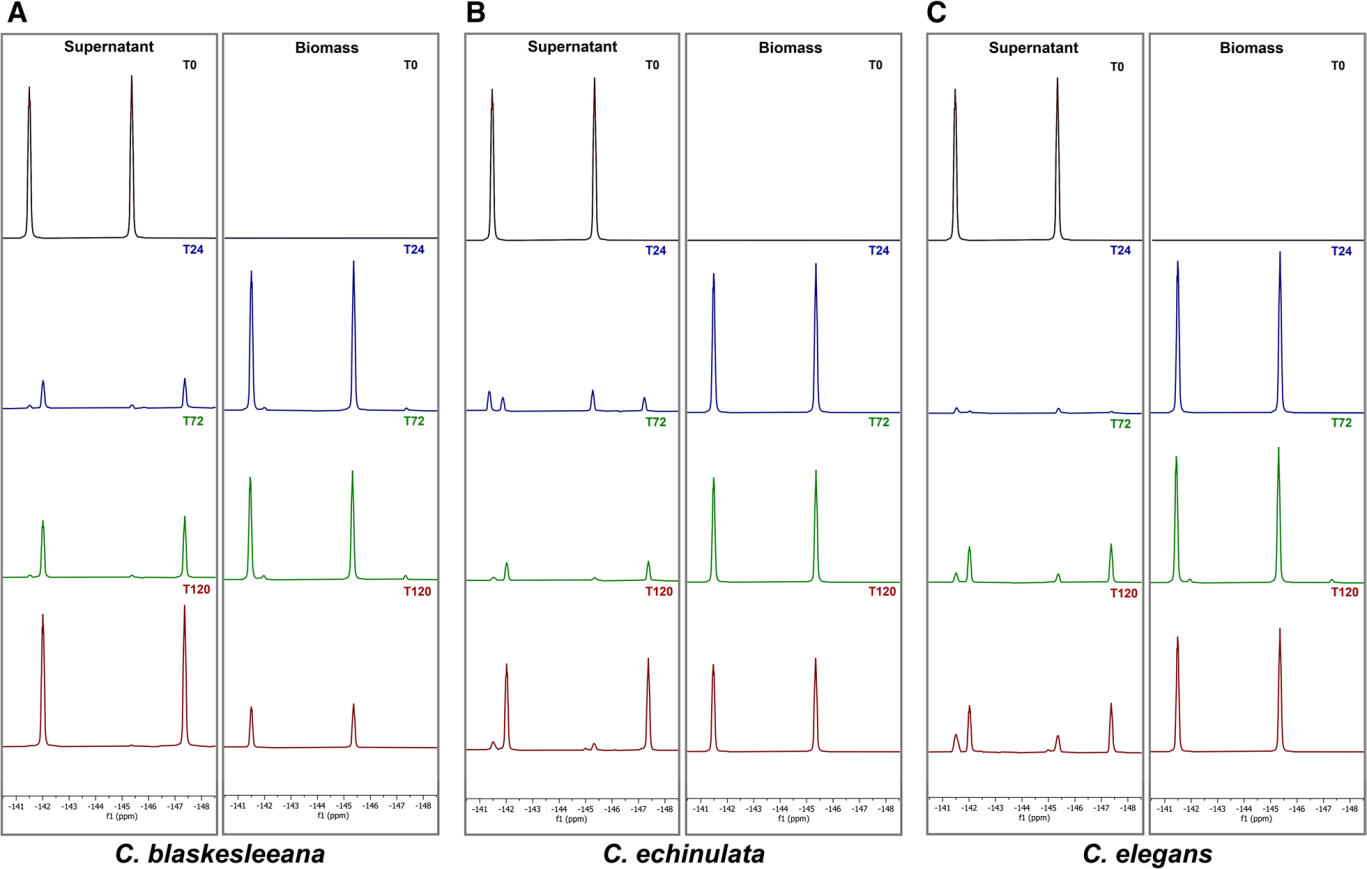
^19^ F NMR spectra of supernatant and biomass extracts of *C. blakesleeana* (**A**), *C. echinulata* (**B**) and *C. elegans* (**C**) after incubation with transfluthrin for 120 h

Three species of *Cunninghamella* (*C. elegans*, *C. blakesleeana* and *C. echinulata*) were cultivated in sabauroud dextrose broth before either transfluthrin or β-cyfluthrin (0.1 mg/ml) was added. The biotransformation of the pyrethroids was monitored over time using ^19^F NMR in the first instance, after the cultures had been extracted with ethyl acetate. Figure 2 shows the decoupled spectra obtained for the supernatant and biomass extracts from each fungus at different time points over 120 h after addition of transfluthrin. Of the four fluorine atoms of the substrate, the two atoms at the *ortho*- positions are equivalent and the two fluorine atoms at the *meta* positions are equivalent, thus the spectrum of the starting compound reveals two resonances (δ − 141.8 and − 145.5 ppm). In the first 24 h after addition, the bulk of the transfluthrin is absorbed by the biomass; however, one main fluorinated metabolite appears in the supernatant of all cultures (δ − 142.0 and − 147.3 ppm), which becomes more prominent as the incubation progresses. Transfluthrin was not transformed in control flasks that were not inoculated with fungus. The integrals and splitting pattern (Additional file 1: Fig S1) indicate that there are the same number of fluorine atoms (4) in the metabolite, thus no defluorination occurred. On the basis of ^19^F NMR analysis of the combined supernatant and biomass extracts (Additional file 1: Fig S2), *C. blakesleeana* appears to most effectively biotransform transfluthrin (78% biotransformed into product), followed by *C. echinulata* (50%) and *C. elegans* is the least effective (20%).

In contrast to transfluthrin biotransformation, ^19^F NMR analyses of culture supernatant and biomass extracts of fungi incubated with β-cyfluthrin revealed that the most prominent resonance was from the starting material (δ − 130.5 ppm), which mostly remained in the biomass of all three species throughout the course of the incubation (Additional file 1: Fig S3). A metabolite was detected (δ − 128.4 ppm) in the supernatants of *C. echinulata* and *C. elegans* and the overall biotransformation was about 30% (Additional file 1: Fig S4). In contrast, no biotransformation of the pesticide was detected in *C. blaskesleeana* cultures.

### Identification of fungal metabolites

**Fig. 3:**
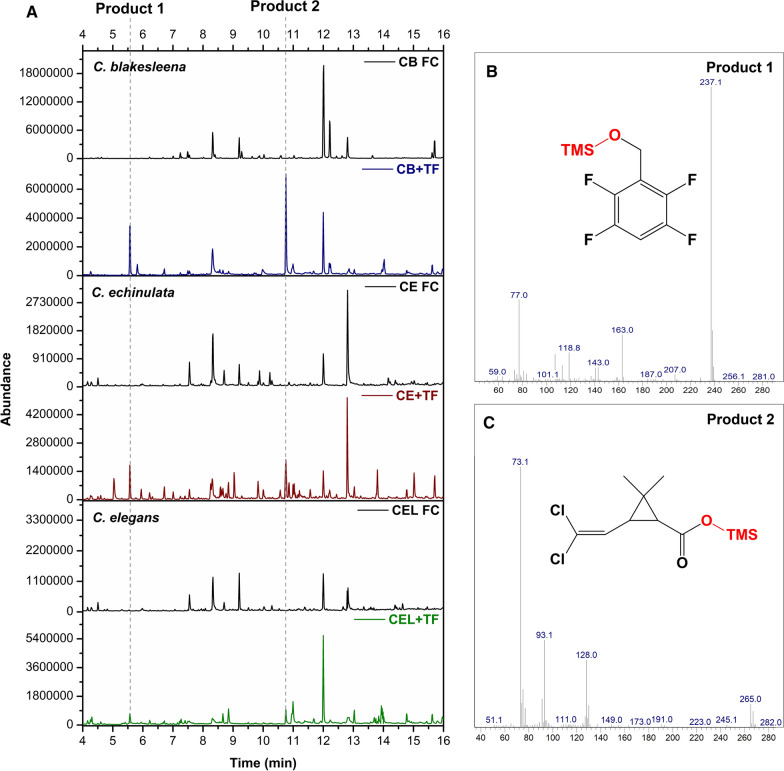
Metabolites from transfluthrin biotransformation. **A** Total ion chromatogram of culture supernatant extracts of fungi showing the production of two metabolites eluting at 5.68 min (Product 1) and 10.76 min (Product 2). The mass spectra of the products are shown in **B**, **C** along with the proposed structures based on comparison of the spectra with those in the NIST database. CB FC: *C. blakesleeana* without transfluthrin; CB + TF: *C. blaskesleeana* plus transfluthrin; CE FC: *C. echinulata* without transfluthrin; CE + TF: *C. echinulata* plus transfluthrin; CEL FC: *C. elegans* without transfluthrin; CEL + TF: *C. elegans* plus transfluthrin

To explore the biodegradation of the pyrethroid pesticides further, the extracts from the cultures were analysed by GC–MS after derivatisation by silylation. Two metabolites were identified in cultures that were incubated with transfluthrin and which were not present in control experiments with fungi that were incubated in the absence of the compound or flasks not inoculated with fungi (Fig. 3). The first metabolite eluted after 5.68 min and its mass spectrum matched that of silylated tetrafluorobenzyl alcohol; the second eluted after 10.76 min and its mass spectrum matched that of silylated dichlorovinyl-2,2-dimethylcyclopropane carboxylic acid. These metabolites would be the expected products upon hydrolysis of the ester bond in transfluthrin. The peak heights observed in the GC–MS analysis of the different fungal extract reflected what was observed in the ^19^F NMR analyses, with *C. blakesleeana* producing more metabolite than *C. echinulata* or *C. elegans*. No other metabolites were detected by GC–MS indicating that the two main products were not biotransformed further by the fungi, which is consistent with the ^19^F NMR analyses which indicated no loss of fluorine from the phenyl ring. Inclusion of the cytochrome P450 inhibitor 1-aminobenzotriazole in the cultures incubated with transfluthrin prevented any biotransformation of the pesticide (not shown), thus it is most likely that *Cunninghamella*’s CYP activity is responsible for the hydrolysis of the ester bond.

**Fig. 4:**
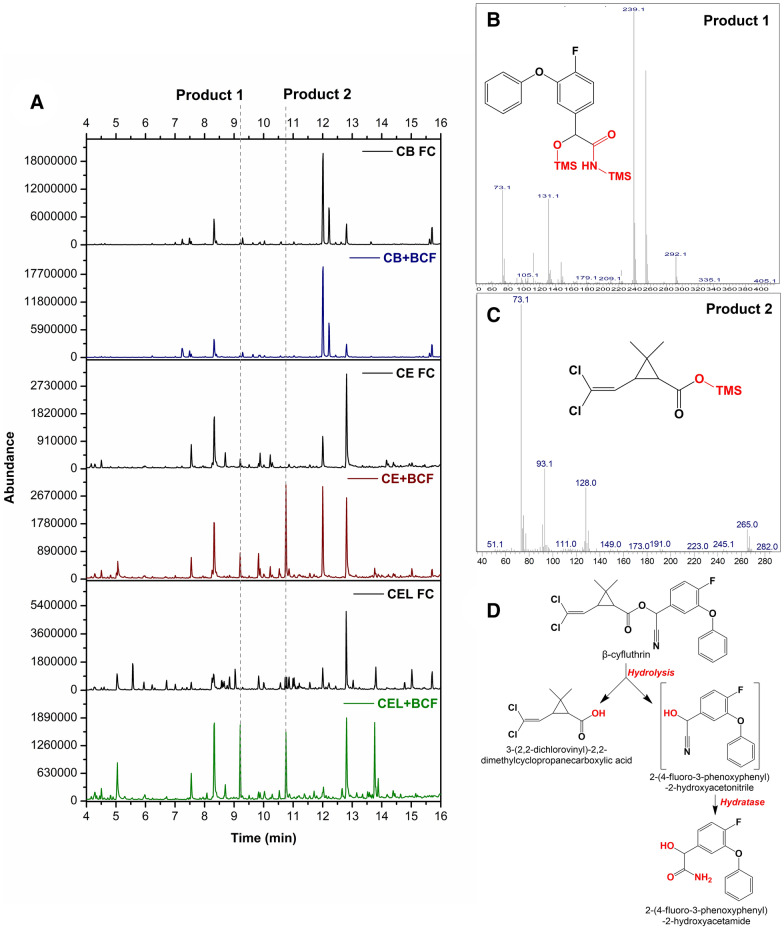
Metabolites from β-cyfluthrin biotransformation **A** Total ion chromatogram of culture supernatant extracts of fungi showing the production of two metabolites eluting at 9.2 min (Product 1) and 10.8 min (Product 2). The mass spectra of the products are shown in **B**, **C** along with the proposed structures based on comparison of the spectra with those in the NIST database. A proposed pathway is shown in (**D**). CB FC: *C. blakesleeana* without β-cyfluthrin; CB + BCF: *C. blaskesleeana* plus β-cyfluthrin; CE FC: *C. echinulata* without β-cyfluthrin; CE + BCF: *C. echinulata* plus β-cyfluthrin; CEL FC: *C. elegans* without β-cyfluthrin; CEL + BCF: *C. elegans* plus β-cyfluthrin

Limited biotransformation of β-cyfluthrin was observed in *C. echinulata* and *C. elegans* by ^19^F NMR (Additional file 1: Fig S4). Even so, the greater sensitivity of GC–MS enabled the detection in these cultures of the same dichlorovinyl-2,2-dimethylcyclopropane carboxylic acid metabolite that was found in transfluthrin experiments (Fig. 4A, C). In addition, another product was observed in the samples eluting at 9.2 min, which is not possible to fully identify, but has an expected molecular ion (M+) of 2-(4-fluoro-3-phenoxy-phenyl)-2-hydroxy-acetamide (Fig. 4A, B). It is conceivable that this could result from the nitrile product of the initial ester hydrolysis that was subsequently hydrated (Fig. 4D). As with transflutrin, no biotransformation products were observed in the presence of the CYP inhibitor.

### Biotransformation of transfluthrin and β-cyfluthrin in rat liver microsomes

**Fig. 5:**
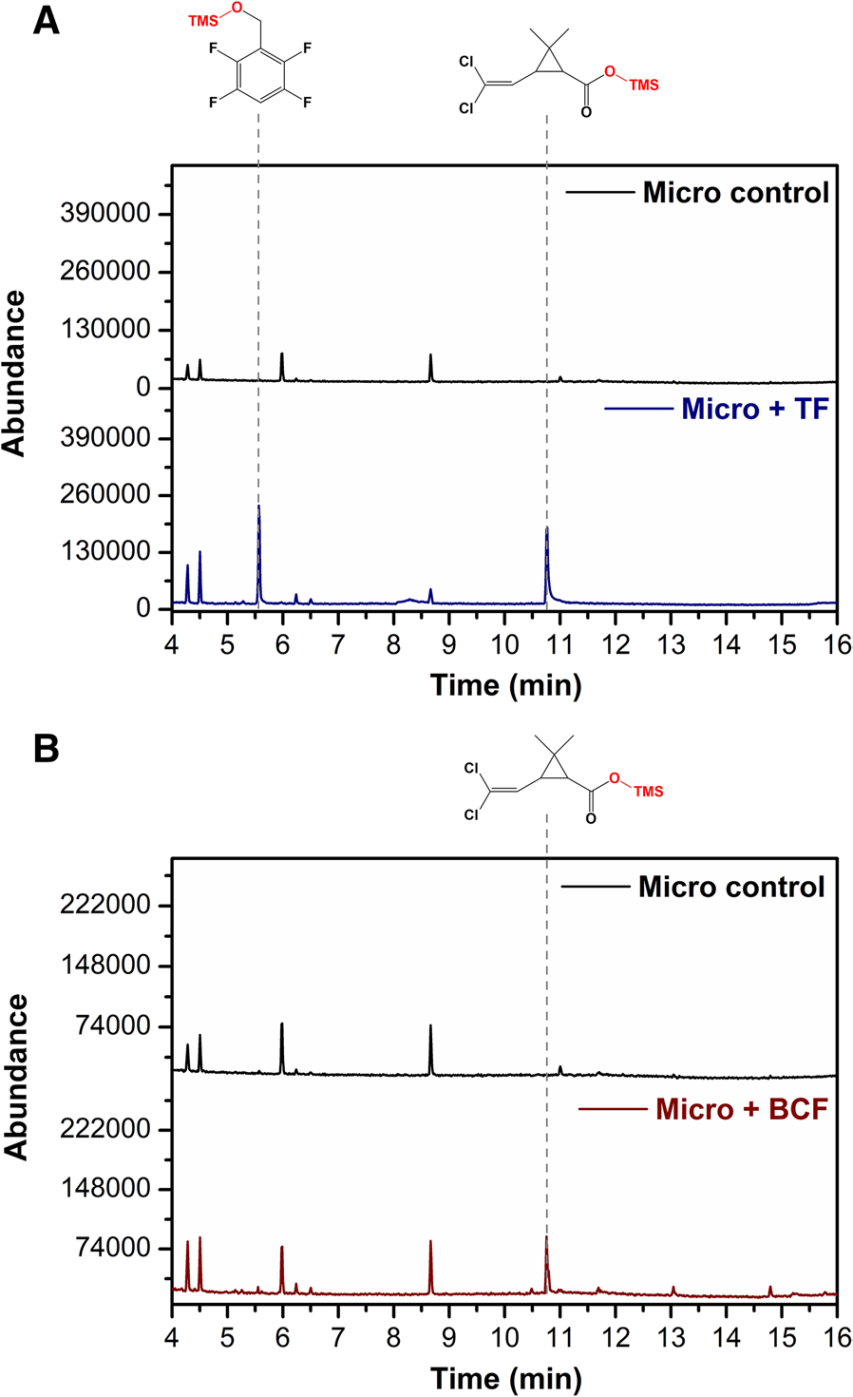
Metabolites detected by GC–MS after microsomal incubation with transfluthrin (**A**) and β-cyfluthrin(**B**)

The pesticides were incubated with rat liver microsomes to determine if the metabolites formed were comparable to those produced in the fungal cultures. Figure 5A shows that upon incubation with transfluthrin two prominent products were formed, which were the same as in the fungal experiments. In the case of β-cyfluthrin, only the chlorinated product was detected (Fig. 5B), suggesting that the fluorinated moiety was not readily detectable using the method presented. Furthermore, the amide product that was detected in the fungal experiments is unique to fungi as it was not observed in the microsomes.

## Discussion

Health and environmental concerns over pyrethroids has led to numerous investigations on the biodegradation of these pesticides in microorganisms, and in particular bacteria (Bhatt et al. 2019). In this paper, we investigated the biotransformation of two fluorinated pyrethroids transfluthrin and β-cyfluthrin in three species belonging to the fungal genus *Cunninghamella*. Whilst β-cyfluthrin is known to be degraded by bacteria including *Brevibacterium aureum* (Chen et al. 2013), *Lysinibacillus sphericus* (Hu et al. 2014) and *Photobacterium ganghwense* (Wang et al. 2019), microbial transformation of transfluthrin has not been evaluated as far as we can tell.

*Cunninghamella* spp. are predominatly used as microbial models of mammalian drug metabolism (Piska et al. 2016) and in this paper we demonstrated that they are also models of mammalian metabolism of pyrethroid pesticides. All three fungi examined, *C. echinulata*, *C. blaskesleeana* and *C. elegans*, biotransformed transfluthrin to the same two metabolites that were detected in rat liver microsome experiments: tetrafluorobenzyl alcohol and dichlorovinyl-2,2-dimethylcyclopropane carboxylic acid. Furthermore, the biotransformation is driven by cytochrome P450 activity as determined by inclusion of a specific inhibitor. This is in contrast to the esterases that are more commonly associated with microbial degradation of pyrethroid insecticides (Bhatt et al. 2020). The difference in the degree of biotransformation, which follows *C. blaskesleeana* > *C. echinulata* > *C. elegans*, also suggests that the CYPs of the three fungi are different in their preference for transfluthrin as a substrate. Human CYPs also differ in their ability to transform pyrethroid pesticides: (Hedges et al. 2020) recently examined the metabolism of five pyrethroids (bifenthrin, λ-cyhalothrin, β-cyfluthrin, cyphenothrin and esfenvalerate) with a range of recombinant human CYPs (2B6, 2C8, 2C9, 2C19, 3A4 and 3A5) and found a wide variation in the ability of the enzymes to transform the pesticides, with CYP2C19 the most active with all of the substrates examined.

*C. echinulata* and *C. elegans*, but not *C. blaskesleeana*, also transformed β-cyfluthrin. The biotransformation of this pesticide was studied previously in the fungus *Trichoderma veride* (Saikia and Gopal 2004) and in that study α-cyano-4-fluoro-3-phenoxy benzyl alcohol, and 3(2,2-dichlorovinyl)-2,2-dimethyl cyclopropanoic acid were determined as the main products. Similar products were detected in *Cunninghamella* spp. except the cyano group was hydrated to form the amide. In rat liver microsomes only the carboxylic acid product was detected. The difference in the biotransformation rates in the three fungi again underscores the CYP substrate specificities in the different species.

The use of fungi to model the metabolism of pesticides is one method to obviate the use of animals in early metabolism study and enables the relatively convenient production of metabolites for further toxicity testing without employing classical organic synthesis approaches that have environmental downsides. In this study we have demonstrated that *Cunninghamella* fungi are appropriate microorganisms to produce mammalian-equivalent metabolites from fluorinated pyrethroid pesticides.

## Supplementary Information

**Additional file 1.** Additional figures.

## Data Availability

Data will be made available on reasonable request.
