# Supplementary material for: Cunninghamella spp. produce mammalian-equivalent metabolites from fluorinated pyrethroid pesticides
Source: AMB Express. 2021 Jul 8;11:101. doi: 10.1186/s13568-021-01262-0 (PMC8266954; doi:10.1186/s13568-021-01262-0)
Supplement: Supplementary file 1 — Additional file 1. Additional figures. [file 13568_2021_1262_MOESM1_ESM.docx]

*AMB Express*

***Cunninghamella* spp. produces mammalian-equivalent metabolites from fluorinated pyrethroid pesticides**

Mohd Faheem Khan and Cormac D. Murphy

UCD School of Biomolecular and Biomedical Science, University College Dublin, Belfield, Dublin 4, Ireland

*Corresponding author [cormac.d.murphy@ucd.ie](mailto:cormac.d.murphy@ucd.ie); phone +353 (0)17162572; fax +353 (0) 17161183

**Supplemental information**


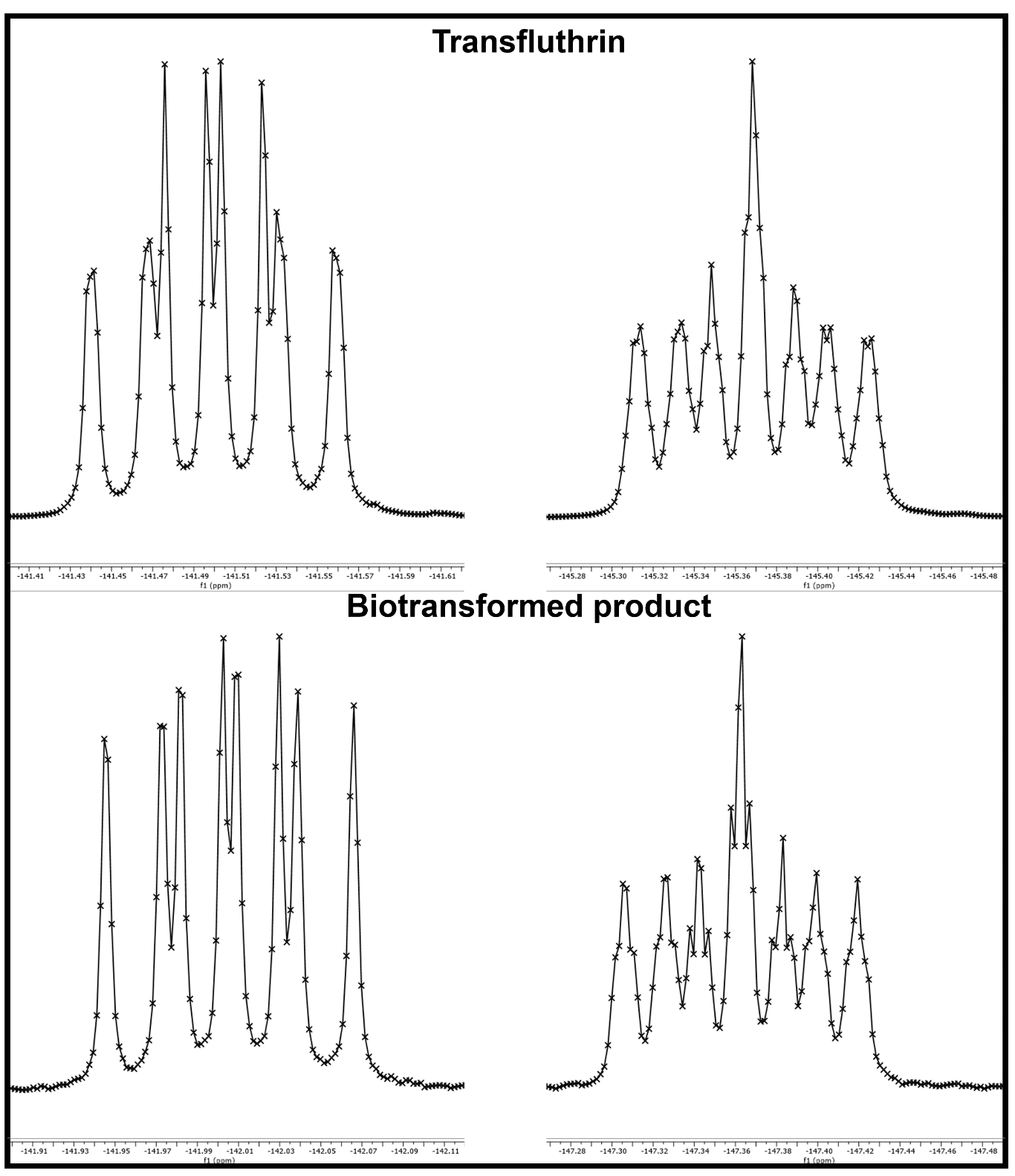


Fig S1. ^1^H-coupled ^19^F NMR spectrum of transfluthrin and the metabolite formed after incubation with *Cunninghamella* spp. The splitting pattern is the same for each compound indicating that no loss of fluorine occurred in the biotransformation reaction.


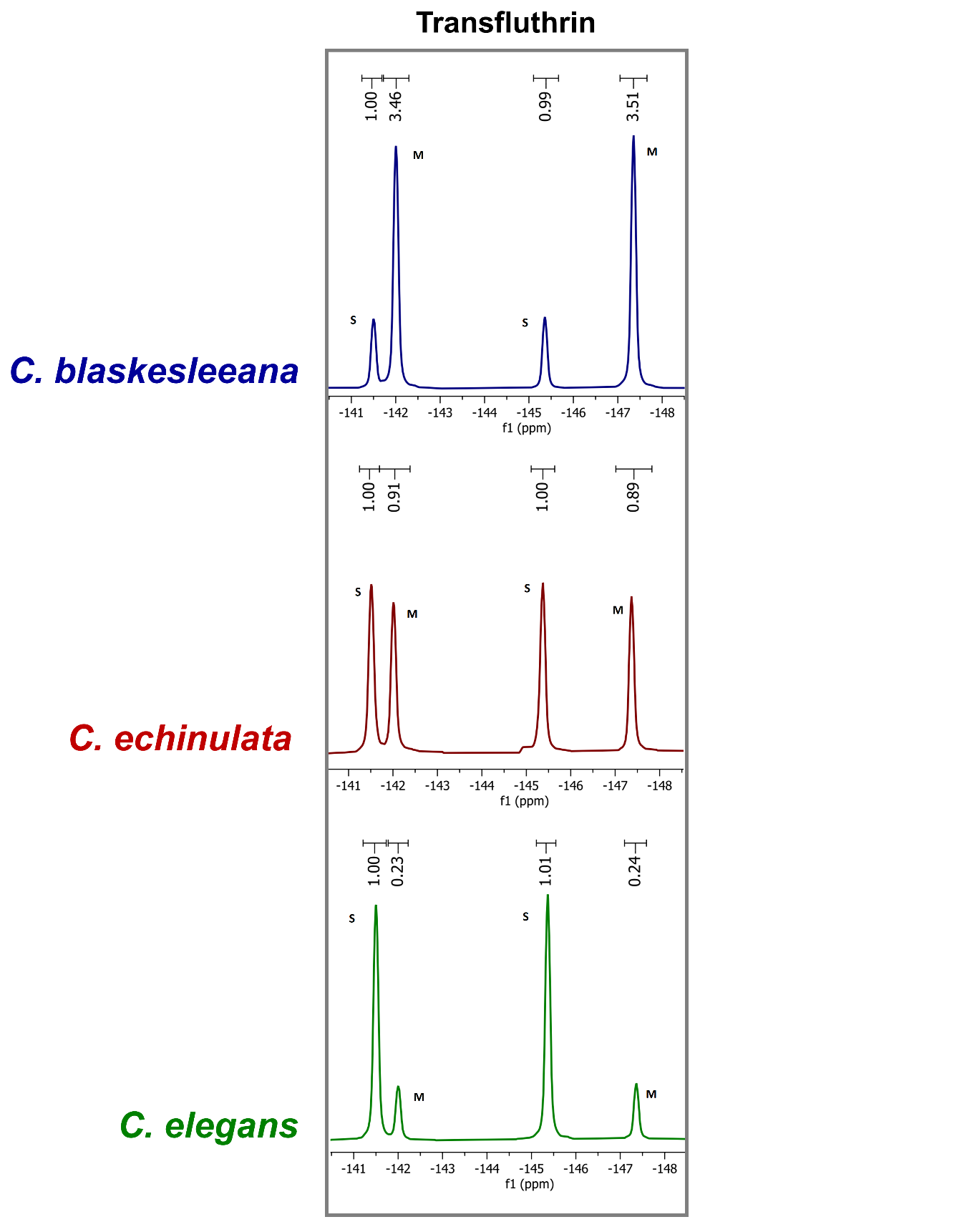


Fig S2. Integration of starting substrate (S) and metabolite (M) peaks in the ^19^F NMR spectrum of the combined extracts from biomass and supernatants collected after 120 h incubation with transfluthrin. Biotransformation of the pesticide was greatest in cultures of *C. blakesleeana* and poorest in *C. elegans* according to the integrals.


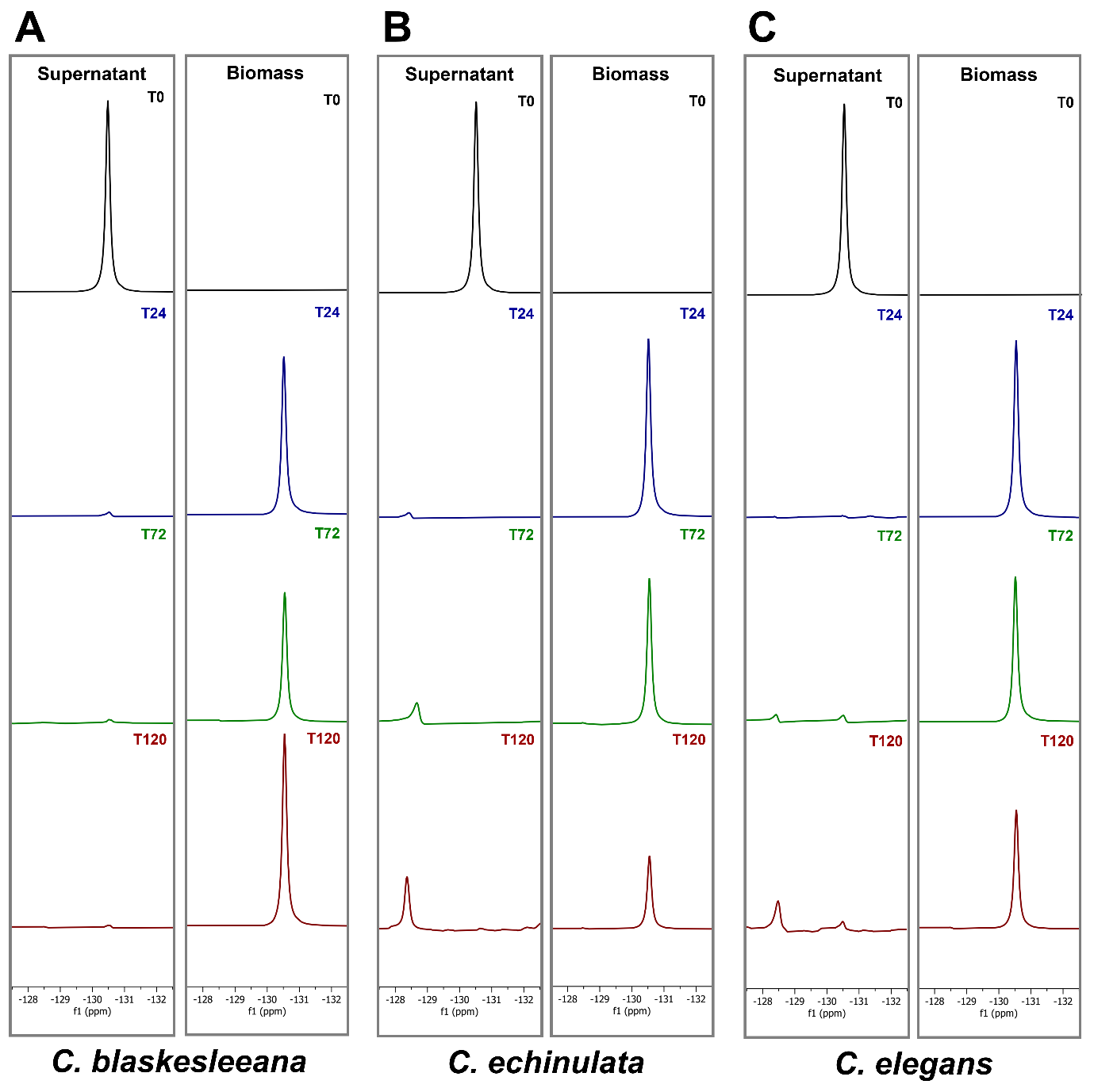


Fig S3. Distribution of β-cyfluthrin and its metabolite in biomass and supernatant.


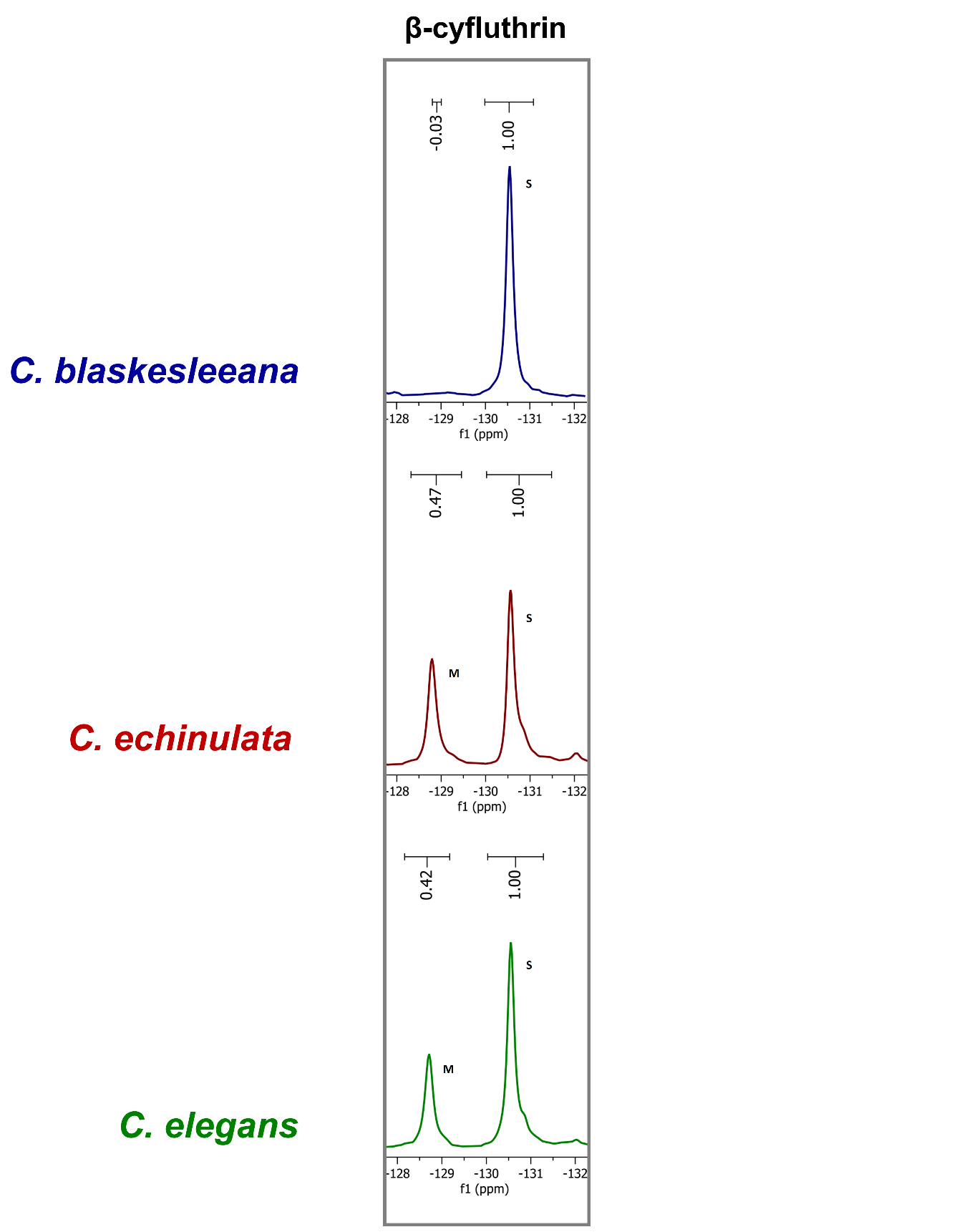


Fig S4. Integration of starting substrate (S) and metabolite (M) peaks in the ^19^F NMR spectrum of the combined extracts from biomass and supernatants collected after 120 h incubation with β-cyfluthrin.
